# The potential of glutamine supplementation in reduced-crude protein diets for chicken-meat production

**DOI:** 10.1016/j.aninu.2024.03.017

**Published:** 2024-05-10

**Authors:** Peter H. Selle, Shemil P. Macelline, Mehdi Toghyani, Sonia Yun Liu

**Affiliations:** a Poultry Research Foundation within the University of Sydney, Camden, NSW 2570, Australia; b Sydney School of Veterinary Science, The University of Sydney, Camden, NSW 2570, Australia; c School of Life and Environmental Sciences, Faculty of Science, The University of Sydney, Camden, NSW 2570, Australia

**Keywords:** Broiler chicken, Glutamate, Glutamic acid, Glutamine

## Abstract

This review explores the potential of including glutamine, a so-called non-essential amino acid, in the formulation of reduced-crude protein (CP) diets for broiler chickens. There is a precedent for benefits when including glycine and serine in reduced-CP diets. Fundamentally this is due to decreases in non-essential amino acid concentrations in reduced-CP diets — an unavoidable consequence of reducing CP without amino acid supplementation. The situation for glutamine is complicated because analysed dietary concentrations are very rarely provided as standard assays do not differentiate between glutamine and glutamate and are reported on a combined basis as glutamic acid. The dietary requirement for glutamic acid is approximately 36.3 g/kg but it is increasingly unlikely that this requirement will be met as dietary CP levels are progressively reduced. Glutamine is an abundant and versatile amino acid and constitutes 50.5 mg/g of whole-body chicken protein and is the dominant free amino acid in systemic plasma where it has been shown to provide 22.6% (139.9 of 620.3 μg/mL) of the total in birds offered 215 g/kg CP, wheat-based diets. In addition to dietary intakes, glutamine biosynthesis is derived mainly from the condensation of glutamate and ammonia (NH_3_) catalysed by glutamine synthetase, a reaction that is pivotal to NH_3_ detoxification. Glutamate and NH_3_ are converted to glutamine by phosphate-dependent glutaminase in the reciprocal reaction; thus, glutamine and glutamate are interchangeable amino acids. However, the rate of glutamine biosynthesis may not be adequate in rapidly growing broiler chickens and exogenous and endogenous glutamine levels are probably insufficient in birds offered reduced-CP diets. The many functional roles of glutamine, including NH_3_ detoxification and maintenance of acid-base homeostasis, then become relevant. Twenty feeding studies were identified where dietary glutamine supplementation, usually 10 g/kg, was evaluated in birds kept under thermoneutral conditions. On balance, the outcomes were positive, but the average dietary CP was 213 g/kg across the twenty feeding studies, which indicates that CP and, in turn, glutamine concentrations would have been adequate. This suggests that glutamine inclusions in reduced-CP diets hold potential and consideration is given to how this may be best confirmed.

## 1 Introduction

The advantages of reduced-crude protein (CP) diets in chicken-meat production are compelling as they range from an attenuation of nitrogen (N) and ammonia (NH_3_) emissions to a reduced dependency on imported, expensive soybean meal in most countries of the world (Greenhalgh et al., 2020a). However, the successful development of reduced-CP broiler diets proves challenging as growth performance is usually compromised, especially when such diets are based on wheat rather than maize. The growth performance of broiler chickens offered wheat-based reduced-CP diets was of an unacceptable standard in Greenhalgh et al. (2020b) and grossly inferior to their maize-based counterparts in Chrystal et al. (2021), which illustrates the disadvantages inherent in reduced-CP broiler diets.

A pivotal breakthrough in the formulation of reduced-CP diets was generated by the Dean et al. (2006) study, which demonstrated the need to consider inclusions of glycine, or glycine equivalents (glycine + 0.7143 × serine), in reduced-CP diets. Subsequently, Siegert and Rodehutscord (2019) suggested that glycine equivalents should range between 11 and 20 g/kg in diets for young broilers. Fundamentally, this indicates that concentrations of the so-called non-essential amino acids, without supplementation, can become inadequate pursuant to dietary CP reductions. This should also apply to glutamine and the outcomes of the Ibrahim et al. (2024) study become relevant as it was suggested that glutamine and asparagine supplementation permitted greater substitutions of peptide-bound with non-bound amino. The substitution of protein-bound amino acids with non-bound (synthetic, crystalline) amino acids (NBAA) is the hallmark of reduced-CP broiler as it allows decreased soybean meal inclusions (Selle et al., 2020a).

Concentrations of glutamine and glutamate in diets and feedstuffs are usually expressed on a combined glutamic acid basis. However, the distinction between glutamine, glutamate and glutamic acid is drawn in this review. Glutamine and glutamate are separate entities; however, glutamine is converted to glutamate by acidic extractions in standard amino acid assays. For this reason, “glutamine” and “glutamate” refer to the amino acid entities specifically and “glutamic acid” refers to both amino acids throughout this paper. Clearly, specific glutamine and glutamate analyses would be preferable and may be completed, following enzymatic hydrolysis, by reverse-phase, high-performance liquid chromatography (Tsao and Otter, 1999). Consequently, there is a paucity of data for specific glutamine and glutamate concentrations in relevant feedstuffs. Glutamine proportions of glutamic acid concentrations ranged from 54.4% in casein, 39.6% in fishmeal, 61.4% in corn grain, 31.8% in meat and bone meal, 47.6% in dehulled soybean meal to 41.9% in sorghum grain as reported by Li et al. (2011). Hou et al. (2019) reported glutamine proportions of 94.2% in wheat flour, 62.3% in corn grain and 45.0% in soybean. Glutamine proportions of 38.6% in corn grain, 59.0% in meat and bone meal, 52.3% in soybean meal and 58.1% in sorghum were tabulated by He et al. (2021). Thus, the balance of glutamine to glutamate concentrations in typical broiler diets is not really known.

Probably only two recommendations for dietary concentrations for glutamic acid have been advanced (Wu, 2014; Maharjan et al., 2021); the average glutamic acid recommendation is 36.3 g/kg, or 288 relative to digestible lysine (100) at 12.63 g/kg. This translates to 15.2 g/kg glutamine (120 relative to lysine) and 21.1 g/kg glutamate (167 relative to lysine), based on the Wu (2014) proposal. However, the validity of these two recommendations has yet to be verified in the context of reduced-CP diets. The successful development of reduced-CP broiler diets is proving a challenge and declares the need for a better understanding of amino acid metabolism (Kidd et al., 2021). Thus, the purpose of this review is to consider the role of glutamine in chicken-meat production in the context of reduced-CP diets as the likelihood is that glutamine merits closer attention in the formulation of reduced-CP diets.

## 2 Glutamine

The interchangeable amino acids, glutamine and glutamate, are “building-blocks” of protein as glutamine represents 50.5 mg/g and glutamate 82.9 mg/g of whole-body protein in broiler chickens, or, collectively, 133.4 mg/g as glutamic acid (Wu, 2014). Alternatively, glutamic acid constitutes 149.1 mg/g of breast meat protein (Hamm, 1981). However, their complex functional roles demand consideration because glutamine and glutamate are vital metabolites as they have a central role in cell metabolism and function (Newsholme et al., 2003). Glutamine is a key link in the carbon metabolism of protein and carbohydrates with the potential to improve nitrogen balance and preserve glutamine concentrations in skeletal muscle (Tapiero et al., 2022). Glutamine metabolism in humans has been thoroughly reviewed by Cruzat et al. (2018) in which glutamine was described as the most abundant and versatile amino acid in the human body. Also, glutamine is integral to intermediary metabolism, interorgan nitrogen exchange via NH_3_ transport between tissues, pH homeostasis and as a precursor for a range of metabolites.

Pursuant to protein digestion in the gut lumen, intestinal uptakes of glutamine and glutamate take place mainly as constituents of di- and tri-peptides (oligopeptides) via the peptide transporter 1, which requires proton donations from the Na^+^/H^+^ exchanger (NHE), to co-transport oligopeptides (Spanier, 2014). In contrast, monomeric or non-protein bound glutamine is absorbed via a range of Na^+^-dependent and Na^+^-independent neutral amino acid transport systems and glutamate intestinal uptakes are via Na^+^-dependent and Na^+^-independent anionic amino acid transporters (Hyde et al., 2003). Thus, intestinal uptakes of “free” or monomeric glutamine and glutamate are via different routes. The intestinal uptakes of NBAA are more rapid than protein-bound amino acids because prior digestion is not required (Wu, 2009).

Following their intestinal uptakes, amino acids must transit through the enterocytes of the gut mucosa to enter the portal circulation and the systemic pool but they may be diverted into anabolic and/or catabolic pathways. The anabolic pathways are entirely necessary as amino acids are required to synthesise proteins to maintain gut integrity, for secretion of mucin and digestive enzymes, and to serve as precursors for other amino acids, polyamines and nucleotides (Wu, 1998). Alternatively, the catabolism of amino acids seems to be an unnecessary and inefficient process. Nevertheless, the gastrointestinal tract consumes about 20% of incoming dietary energy to digest and absorb nutrients (Cant et al., 1996) and amino acids are catabolised to meet this demand. Specifically in poultry, Watford et al. (1979) found that glucose, glutamine and glutamate were the preferred energy substrates in isolated avian enterocytes. Similarly, Porteous (1980) reported that glucose, glutamine and glutamate were the only individual substrates to stimulate respiration in isolated avian enterocytes. However, He et al. (2022) concluded that glutamate was the principal energy source for enterocytes in broiler chickens. Concentrations of 5.0 mmol/L of glutamate, glucose, aspartic acid and glutamine were oxidised in enterocytes to yield 48.8, 17.7, 14.3 and 8.36 CO_2_ units, respectively, in 42-d-old chickens. Thus, the oxidation of glutamate exceeded that of glutamine by a factor of 5.8, aspartate by a factor of 3.4 and glucose by a factor of 2.8.

Glutamine is classified as non-essential amino acid in poultry diets because it can be synthesised endogenously by broiler chickens. In addition to dietary sources, glutamine is derived mainly from the condensation of glutamate and NH_3_, a reaction catalysed by glutamine synthetase. In the reciprocal reaction, glutamine is converted to glutamate and N, which is catalysed by a phosphate-dependent glutaminase. However, glutamate biosynthesis also stems from intermediate metabolites of the citric acid cycle (Cruzat et al., 2018). Thus, glutamine and glutamate are interconvertible amino acids, but their metabolic roles are quite different as glutamate is the major N donor in metabolism; whereas glutamine assimilates N as NH_3_. The renal metabolism of glutamine is pertinent as the kidney takes up glutamine and metabolizes it to NH_3_, but this process is sensitive to pH and serves to maintain acid-base homeostasis. Metabolic acidosis induces increases in the renal uptake and breakdown of glutamine resulting in ammoniagenesis from increased glutaminase activity in order to buffer urine pH and maintain acid-base homeostasis (Van de Poll et al., 2004).

The interorgan metabolism and nutritional impacts of dietary glutamine and glutamate in poultry have been extensively reviewed by He et al. (2021) who contended that their biosynthesis was insufficient to support maximum lean tissue gain and feed efficiency. In addition, glutamine and asparagine metabolism was investigated by Coon and Balling (1984) who suggested that asparagine may be more important than glutamine in regulating acidosis in broiler chickens. The influence of glutamine on intestinal physiology, immunology and microbiology in broilers was reviewed briefly by Bortoluzzi et al. (2018). These authors emphasised the importance of glutamine in relation to intestinal structure and function. This is reflected in the Maiorka et al. (2016) study, where the addition of 10 g/kg glutamine to a 220 g/kg CP, maize-soy diet was examined. Glutamine significantly increased villus height in the duodenum, jejunum and ileum in broiler chickens at 7 d of age, which illustrates the high requirements that proliferating enterocytes have for glutamine.

It is then relevant that the impacts of CP reductions in maize- and wheat-based diets were investigated in a series of seven studies (Chrystal et al., 2020a,b,c, 2021; Greenhalgh et al., 2020b, 2022; Yin et al., 2020). The CP of maize-based diets were reduced from an average of 207 to 165 g/kg, which resulted in analysed dietary concentrations of glutamic acid falling from 35.5 to 25.0 g/kg. The CP of wheat-based diets were reduced from 217 to 164 g/kg which resulted in a decline in glutamic acid from 42.9 to 31.5 g/kg. Given that Wu (2014) and Maharjan et al. (2021) recommended dietary glutamic acid concentrations of 36.3 g/kg, this indicates that, without supplementation, dietary CP reductions of this order will result in inadequate glutamic acid concentrations and implies that both glutamine and glutamate may be insufficient. Free glutamine and glutamate concentrations in both portal and systemic plasma were determined in birds offered wheat-based diets with CP contents of 215 and 165 g/kg CP in Yin et al. (2020). On average, glutamine (164.7 μg/mL) made up 85.9% and glutamate (27.1 μg/mL) 14.1% of total glutamic acid. The dominance of free glutamine in plasma may reflect its role in inter-organ N transport as the N component of glutamine (191.7 g/kg) is double that of glutamate (95.2 g/kg).

## 3 Reduced-crude protein diets

A typical reduced-CP diet contains higher feed grain inclusions, less soybean meal, but elevated inclusions of NBAA. Moderate reductions of up to 30 g/kg CP are reasonably well tolerated by broiler chickens but more tangible reductions may compromise growth performance and increase fat deposition. However, Baker (2009) cautioned that “there are limits to how much intact protein can be replaced by free amino acids in terms of achieving maximal weight gain and feed efficiency of broiler chicks”. The likelihood is that increasing NBAA inclusions generate post-enteral amino acid imbalances (Harper, 1959), simply because their intestinal uptakes are more rapid than protein-bound amino acids. The intestinal uptakes of non-bound lysine HCl and DL-methionine were more rapid than protein-bound amino acids in broiler chickens offered standard CP diets in Liu et al. (2013) and similar findings for DL-methionine and protein-bound methionine were reported by Zamani et al. (2021). Ideally, amino acids are incorporated into protein, but amino acids surplus to requirements for protein synthesis are rapidly catabolised (Brosnan, 2003); indeed, postprandial oxidative losses of NBAA have been shown to exceed those of protein-bound amino acids in rats and humans (Nolles et al., 2009). The catabolism of amino acids generates NH_3_ systemically, which is inherently toxic (Stern and Mozdziak, 2019) and poultry are probably more vulnerable to NH_3_ toxicity than mammalian species (Wilson et al., 1968).

Four amino acids glutamate, glutamine, glycine and serine (or glycine equivalents) are integral to the detoxification of NH_3_ and its elimination as uric acid in broiler chickens. Hakvoort et al. (2017) contended that glutamine synthetase is pivotal to NH_3_ detoxification because it catalyses the reaction in which NH_4_^+^ and glutamate are condensed to form glutamine in the liver as depicted in the Minet et al. (1997) equation:

Glu + NH_4_^+^ + ATP + Mg^2+^ ⇒ Gln + ADP + Pi.

Glutamine generated by this reaction may enter the Krebs uric acid cycle, whereby NH_3_–N is eliminated, as uric acid-N in urine. Importantly, there is an obligatory glycine requirement for the Krebs uric acid cycle as one mole of glycine is demanded for each mole of uric acid excreted (Salway, 2018). The synthesis and excretion of uric acid to void NH_3_–N in urine generates an energy cost of at least 64.7 kJ/g N excreted as uric acid in poultry (Van Milgen, 2021); thereby, contributing to the “costs of deamination” (Selle et al., 2020b).

However, if the capacity of birds to detoxify NH_3_ and eliminate uric acid is compromised, plasma NH_3_ concentrations are increased. Associations between elevated plasma NH_3_ concentrations and depressed growth performance in broiler chickens has been evident in four studies (Aguihe et al., 2022; Namroud et al., 2008; Ospina-Rojas et al., 2013, 2014). It is then evident that glutamine is integral to both NH_3_ detoxification and uric acid elimination.

## 4 Acid-base homeostasis, glutamine and ammonia

Acid-base homeostasis in broiler chickens was reviewed by Mushtag and Pasha (2013), in which a narrow dietary electrolyte balance from 150 to 250 mEq/kg was recommended. Also, attention was drawn to the following Mongin (1981) equation:

(anions – cations) _(in)_ + H^+^_(endogenous)_ – (anions – cations) _(out)_ = 0.

This equation indicates that endogenous acid production (H^+^_(endogenous)_) should be taken into account in the maintenance of acid-base homoeostasis. Endogenous acid mainly arises from the metabolism of dietary proteins as the metabolism of sulphur-containing amino acids and cationic amino acids (arginine, histidine lysine) are common sources of endogenous acid production (Poupin et al., 2012). Dietary CP reductions from 222 to 165 g/kg increased lysine HCl inclusions from 1.60 to 8.12 g/kg in maize-based diets and from 2.36 to 9.72 g/kg in wheat-based diets in Chrystal et al. (2021), which would increase the dietary anionic minus cationic differential; thus, reduced-CP broiler diets might induce metabolic acidosis. There was some evidence of a compensated metabolic acidosis in the Ibrahim et al. (2024) study, where dietary soy protein isolate inclusions were reduced from 80 to 0 g/kg in a stepwise manner and replaced by a NBAA blend. The blend matched the amino acid composition of the soy protein isolate except for glutamine, glutamate, asparagine and aspartate. The experimental diets ranged from 154 to 167 g/kg CP and NBAA blend inclusions from 0 to 46.48 g/kg. Substitutions of the soy protein isolate with the NBAA blend ranged from 0% to 25%, 50%, 75% and 100% and tandem additions glutamine and asparagine increased from nil to 6.64 and 3.78 g/kg, respectively. Weight gain and feed efficiency from 7 to 21 d post-hatch were not influenced by the 25% substitution nor by the 50% substitution with the tandem additions but were depressed by higher substitutions. While not conclusive, Ibrahim et al. (2024) suggested that tandem additions of glutamine and asparagine permitted higher NBAA inclusions without influencing acid–base homeostasis based on determined blood parameters related to acid-base balance which indicated a compensated metabolic acidosis.

Patience (1990) contended that amino acid metabolism and acid-base homeostasis are closely intertwined in animal nutrition and nominated glutamine as the primary amino acid involved in renal ammonia genesis, a process intimately related to acid excretion. Given the inherent toxicity of NH_3_, it is not surprising that sub-clinical NH_3_ toxicity or “NH_3_ overload” may compromise the growth performance of broiler chickens offered reduced-CP diets. However, the capacity of the kidneys to generate NH_3_ enables the kidneys to excrete an acid load and thereby maintain acid-base balance. This is because the provision of a urinary buffer in the form of NH_4_^+^ enhances H^+^ secretion and excretion; therefore, an increase in renal NH_4_^+^ production, and a in urinary buffering capacity results in an increase in acid excretion (Tannen, 1983). Importantly, Tannen (1983) contended that the conversion of glutamine to NH_4_^+^ accounts for enhanced renal NH_4_^+^ production under conditions of metabolic acidosis. More recently, Weiner and Verlander (2019) reasoned that NH_3_ metabolism plays a critical role in acid-base homeostasis and that NH_3_ in kidneys is selectively transported either into the urine or the renal vein but that only the proportion of NH_3_ excreted in urine contributes to acid-base homeostasis. Interestingly, the addition of 30 g/kg L-cysteine to a nutritionally adequate maize-soy diet was shown to induce a lethal metabolic acidosis in broiler chickens by Dilger and Baker (2008).

The interorgan metabolism of glutamine and its role in acid-base balance in humans was specifically reviewed by Taylor and Curthoys (2004). Normally, the primary sites of glutamine synthesis and release are muscle and adipose tissue and glutamine is mainly utilised by the small intestine and liver. However, in the event of metabolic acidosis, the kidney becomes the major site of glutamine extraction and catabolism. This process generates NH_4_^+^ that is excreted in the urine to facilitate the clearance of acids and bicarbonate ions that enter the circulation to partially compensate the acidosis. The increased renal utilisation of glutamine is balanced by increased releases from muscle and liver and decreased utilisation in the intestine. Decreases in bicarbonate concentrations cause metabolic acidosis and catabolism of dietary and endogenous proteins generate excess acid in the form of sulphate and phosphate ions. Finally, Craan et al. (1982) investigated the capacity of broiler chickens to adapt to metabolic acidosis, which was induced by the daily administration of 0.75 g/kg ammonium chloride via stomach tube for six days. Ammonium chloride generated a decline in plasma bicarbonate concentrations of 62.2% (8.7 versus 23.0 mmol/L) and plasma pH fell from 7.52 to 7.17.

It is evident in the above section that glutamine, NH_3_ and acid-base homeostasis are intricately connected although the complicated mechanisms have yet to be described fully. Nevertheless, this adds support to the Ibrahim et al. (2024) study which suggests high NBAA in broiler diets can cause metabolic acidosis and it is even possible that renal ammoniagenesis contributes to elevated plasma NH_3_ concentrations.

## 5 Responses to glutamine supplementation

Numerous studies have been completed where the impacts of glutamine supplementation upon diets offered to broiler chickens under conditions of stress, including high temperatures, coccidiosis and disease, have been assessed. The impacts of glutamine in birds subjected to heat stress have been systematically reviewed by Ncho et al. (2023) who concluded that glutamine supplementation effectively mitigates the deleterious effects of heat-stress by enhancing the antioxidant status. However, only impacts of dietary glutamine supplementation on the performance of broilers kept under thermoneutral conditions are considered in this review, and other growth performance outcomes from 20 relevant studies identified are summarised in Table 1.

**Table 1:** Effects of glutamine supplementation of diets on growth performance in broiler chickens kept under thermoneutral conditions.

Reference	Description	Outcomes
Sakamoto et al. (2006)	Maize-soy diets CP not stated 1 to 41 d post-hatch	Glutamine at 10 g/kg increased weight gain by 0.51% (2579 vs. 2566 g/bird) Glutamine at 10 g/kg improved FCR by 0.63% (1.736 vs. 1.747) Note: Birds received glutamine for the first 14 d only
Bartell and Batal (2007)∗	Maize-soy diets 225 g/kg CP 1 to 21 d post-hatch	Glutamine at 10 g/kg increased weight gain by 9.21% (771 vs. 706 g/bird; *P* < 0.05) Glutamine at 10 g/kg improved FCR by 3.30% (1.639 vs. 1.695) Glutamine at 40 g/kg depressed weight gain and FCR by 10.2 % and 1.69%, respectively
Murakami et al. (2007)∗	Maize-soy diets 215 g/kg CP 1 to 14 d post-hatch	Glutamine at 10 g/kg increased weight gain by 1.02% (397 vs. 393 g/bird) Glutamine at 10 g/kg improved FCR by 0.24% (1.273 vs. 1.276)
Soltan (2009)∗	Maize-soy diets 221, 202, and 191 g/kg CP 1 to 42 d post-hatch	Glutamine at 10 g/kg increased weight gain by 9.33% (2244 vs. 2052 g/bird; *P* < 0.05) Glutamine at 10 g/kg improved FCR by 5.41% (1.75 vs. 1.85) Growth performance was depressed by glutamine at 15 and 20 g/kg
Fasina et al. (2010)	Maize-soy diets 220 g/kg CP 1 to 14 d post-hatch	Glutamine at 10 g/kg increased weight gain by 10.3% (352 vs. 319 g/bird; *P* < 0.05) Glutamine at 10 g/kg improved FCR by 2.86% (1.256 vs. 1.293; *P* < 0.05) Calculated dietary glutamine increased from 33.3 to 41.9 g/kg
Ebadiasl (2011)^1^	Wheat-soy diets 191 g/kg CP 1 to 35 d post-hatch	Glutamine at 5 and 10 g/kg increased body weight by 3.65% (2073 vs. 2000 g/bird) Glutamine at 5 g/kg depressed FCR by 1.19% (1.70 vs. 1.68) Glutamine at 10 g/kg improved FCR by 2.38% (1.64 vs. 1.68)
Martinez et al. (2012)∗	Maize-soy diets 215 g/kg CP 1 to 21 d post-hatch	Glutamine at 20 g/kg increased weight gain by 3.09% (156.8 vs. 152.1 g/bird) Glutamine at 20 g/kg improved FCR by 4.15% (1.015 vs. 1.059) at 7 d post-hatch Improvements were not observed at 21 d post-hatch
Moghaddam and Alizadeh-Ghamsari (2013)^2^^,^∗	Maize-soy diets 219 g/kg CP 1 to 42 d post-hatch	Glutamine at 10 g/kg increased gain by 10.6% (55.32 vs. 50.00 g/bird; *P* < 0.05) Glutamine at 10 g/kg improved FCR by 5.70% (1.82 vs. 1.93) Glutamine at 15 g/kg tended to depress growth performance
Nascimento et al. (2014)∗	Maize-soy diets 221, 211, 197, and 183 g/kg CP 1 to 42 d post-hatch	Glutamine at 15 g/kg increased weight gain by 2.11% (2900 vs. 2840 g/bird) Glutamine at 15 g/kg improved FCR by 2.07% (1.609 vs. 1.643)
Szabó et al. (2014)^3^	Not stated 228 and 200 g/kg CP 18 to 39 d post-hatch	Glutamine at 5 g/kg decreased weight gain by 1.62% (548 vs. 557 g/bird) Glutamine at 5 g/kg compromised FCR by 2.17% (1.88 vs. 1.84)
Luquetti et al. (2016)	Maize-soy diets 220 g/kg CP 1 to 28 d post-hatch	Glutamine at 10 g/kg decreased weight gain by 0.52% (1331 vs. 1338 g/bird) Glutamine at 10 g/kg improved FCR by 1.36% (1.45 vs. 1.47)
Ribeiro et al. (2015)	Maize-sorghum-soy diets 244, 221, and 185 g/kg CP 1 to 42 d post-hatch	Glutamine at 4.0 g/kg increased weight gain by 4.50% (2556 vs. 2446 g/bird) Glutamine at 4.0 g/kg improved FCR by 2.53% (1.659 vs. 1.702)
Namroud et al. (2017)∗	Maize-soy diets 234 g/kg CP 1 to 14 d post-hatch	Glutamine at 10 g/kg increased weight gain by 8.23% (1184 vs. 1094 g/bird; *P* < 0.05) Glutamine at 10 g/kg improved FCR by 3.67% (1.285 vs. 1.334; *P* < 0.05)
Xue et al. (2018)^4^^,^∗	Wheat-barley-soy diets 241, 224, and 203 g/kg CP 1 to 35 d post-hatch	Glutamine at 10 g/kg increased weight gain by 4.36% (2584 vs. 2476 g/bird; *P* < 0.05) Glutamine at 10 g/kg improved FCR by 1.33% (1.336 vs. 1.354; *P* < 0.05)
Abdulkarimi et al. (2019)∗	Maize-soy diets 203, 180 g/kg CP 7 to 42 d post-hatch	Glutamine at 5 g/kg increased weight gain by 15.4% (1430 vs. 1239 g/bird; *P* < 0.05) Glutamine at 5 g/kg improved FCR by 5.78% (2.070 vs. 2.197) Glutamine at 10 g/kg improved weight gain by 3.07% and FCR by 4.69%.
Barekatain and Toghyani (2019)	Wheat-sorghum-soy diets 226, 207, and 195 g/kg CP 1 to 35 d post-hatch	Glutamine at 10 g/kg decreased weight gain by 0.63% (2836 vs. 2854 g/bird) Glutamine at 10 g/kg depressed FCR by 0.64% (1.406 vs. 1.397)
Barekatain et al. (2019)	Wheat-sorghum-soy diets 194 and 177 g/kg CP 7 to 42 d post-hatch	Glutamine at 10 g/kg decreased weight gain by 7.17% (2137 vs. 2302 g/bird) Glutamine at 10 g/kg compromised FCR by 0.93% (1.08 vs. 1.07)
Carvalho et al. (2020)	Maize-soy diets 229 g/kg CP 7 to 35 d post-hatch	Glutamine at 10 g/kg decreased weight gain by 4.58% (137.5 vs. 144.1 g/bird) Glutamine at 10 g/kg improved FCR by 0.13% (1.519 vs. 1.521)
Karamik and Kop-Bozbay (2020)∗	Maize-soy diets 219 and 198 g/kg CP 1 to 42 d post-hatch	Glutamine at 10 g/kg increased weight gain by 0.39% (2571 vs. 2561 g/bird) Glutamine at 10 g/kg improved FCR by 2.17% (1.80 vs. 1.84) Note: Birds received glutamine for the first 7 d only
Wu et al. (2020)∗	Maize-wheat-soy diets 229 and 215 g/kg CP 1 to 42 d post-hatch	Glutamine at 10 g/kg increased weight gain by 6.72% (106.69 vs. 99.97 g/bird) Glutamine at 10 g/kg improved FCR by 6.57% (1.85 vs. 1.98) *P* < 0.05

“*P* < 0.05” denotes that the response was statistically significant.

1 Calculated glutamic acid in control diet 43 g/kg.

2 Mean calculated dietary glutamic acid concentrations 38.4 g/kg.

3 Mean calculated dietary glutamic acid concentrations 39.3 g/kg.

4 Estimated mean glutamine dietary concentrations 6.4 g/kg.

∗ Positive responses in small intestinal gut morphology reported.

Across the 20 feeding studies the mean dietary CP concentration was 213 g/kg and the average feeding period was from 2.75 to 33.2 d post-hatch. The 2022 Ross nutrition specifications call for a weighted average dietary CP of 214 g/kg for this feeding period; thus, the dietary CP levels in 20 feeding studies were presumably adequate. It is reasonable to assume that glutamine concentrations would have been sufficient given the ostensibly adequate dietary CP concentrations. Calculated dietary specifications for glutamic acid were reported in only three studies, which are included in the footnotes of Table 1. The average glutamic acid specification was 40.2 g/kg, which exceeds the 36.3 g/kg recommendation of Wu (2014) and Maharjan et al. (2021). Also, calculated glutamine concentrations were 33.3 and 41.9 g/kg in Fasina et al. (2010), which also appear adequate.

In Table 1, the average dietary glutamine inclusion was 9.95 g/kg, which generated average improvements in weight gain of 3.25% and in FCR of 2.61%. However, responses ranged from −7.17% to +15.4% in weight gain and from +2.17% to −11.0% in FCR; nevertheless, 15 studies reported positive weight gain responses and 17 studies observed improvements in FCR. These studies would have been more instructive had glutamine concentrations in the basal diets been determined. However, analysed dietary amino acid concentrations were not reported in any of the studies.

In addition to growth performance, positive responses to glutamine in small intestinal gut morphology were reported in 11 of the 19 studies, which are identified in Table 1. For example, 10 g/kg glutamine significantly increased villus height by 6.68% (1357 versus 1272 μm) and decreased crypt depth by 7.01% (160.4 versus 172.5 μm) resulting in an expansion of the villus height to crypt depth ratio from 7.38 to 8.46 at 42 d post-hatch in Wu et al. (2020). Similar significant responses were also observed at 21 d post-hatch. Moreover, 10 g/kg glutamine increased the average apparent ileal digestibility coefficients of 17 amino acids by 9.52% (0.782 versus 0.714) in the Wu et al. (2020) study. The most pronounced responses (>14.5%) were observed for cysteine, glutamic acid, threonine and methionine. Also, Namroud et al. (2017) reported that 20 g/kg glutamine significantly increased apparent ileal digestibility coefficients of lysine (7.05%), arginine (6.90%) and isoleucine (3.53%) but decreased glutamic (4.35%) and aspartic (7.95%) acids. Presumably, the positive amino acid digestibility responses to glutamine stem from enhanced small intestinal gut morphology and function.

Apart from the tabulated studies, Li et al. (2010) compared 10 g/kg AminoGut™, a proprietary blend of glutamine and glutamate, with 10 g/kg glutamine and 10 g/kg glutamate. Both glutamine and the proprietary blend significantly increased glutamine plasma levels and both treatments significantly increased fractional rates of protein synthesis in breast and leg muscle by four percentage units. In contrast, glutamic acid did not influence any of the assessed parameters. Li et al. (2010) concluded that dietary supplementation with glutamine individually, or as a component of AminoGut, promoted growth through stimulating muscle protein synthesis. The increased glutamine plasma levels in response to glutamine, but not glutamate, is consistent with the findings of He et al. (2022), who concluded that glutamate was the principal energy substrate for enterocytes in broiler chickens and the oxidation of glutamate exceeded that of glutamine by a 6-fold factor in the gut mucosa. As glutamate is more likely to be catabolised extensively in the gut mucosa compared to glutamine, it follows that dietary supplementation with glutamine per se is likely to be more effective than glutamate.

In relation to glutamine supplementation stimulating muscle protein synthesis, Rennie et al. (1989) suggested that glutamine-sodium cotransporters in muscle membranes regulate the intracellular glutamine pool and are involved in the anabolic effects of glutamine in promoting protein accretion, with a smaller effect in reducing protein degradation. Glutamine was found to have an overall anabolic effect in isolated avian skeletal muscle by Wu and Thompson (1990) and data in Watford and Wu (2005) implies that there is a positive relationship between intramuscular glutamine concentrations and protein synthesis in chickens. If glutamine does promote protein synthesis in rapidly growing broiler chickens it is possible that systemic NH_3_ levels arising from protein turnover would be ameliorated.

Glutamine supplementation at 5 and 10 g/kg was evaluated in 211 g/kg CP, maize-based diets, which were offered to Qiandongnan Xiaoxiang chickens, a small native Chinese breed in Zhang et al. (2022). As a main effect, 10 g/kg glutamine additions significantly improved weight gain by 6.53% (42.11 versus 39.53 g/d), feed intake by 9.05% (13.25 versus 12.15 g/d) and FCR by 2.76% (3.17 versus 3.26) to 42 d post-hatch. These findings may not apply to commercial broiler chickens, but they do illustrate the benefits of glutamine supplementation in poultry.

The CP adequacy of diets used in the tabulated studies poses the question as to the magnitude of responses to glutamine that might be observed with reduced-CP diets containing marginal or insufficient levels of glutamine. This question was partially addressed by Kriseldi et al. (2017) as the CP of the maize-soy diets was reduced by 24 g/kg, which is moderate, and glutamine was primarily used as a N source because of its high N content of 192 g/kg. However, the researchers concluded that glutamine improved FCR during the finisher phase (29 to 41 d post-hatch) and supplementation of glutamine and glycine in tandem supported better total breast meat weight and yield than either amino acid individually. These outcomes are not conclusive, but they do indicate that evaluations of glutamine in reduced-CP diets are justified.

## 6 Future directions

The inclusion of glutamine in reduced-CP broiler diets would appear to hold promise and merits investigation. Therefore, it should prove instructive to employ an equilateral triangle response surface design based on reduced-CP, wheat-based diets (165 g/kg CP) in which concentrations of glutamine and glutamate are specifically determined. A dietary CP concentration of 165 g/kg would represent a notional CP reduction of 45 g/kg over a feeding period from 7 to 35 d post-hatch. The three apical diets would be supplemented with either (1) 20 g/kg glutamine, (2) 20 g/kg glutamate and (3) 25 g/kg glycine equivalents. Promising outcomes for 10 g/kg glutamine have been reported; therefore 20 g/kg inclusions of glutamine and glutamate in apical diets appear appropriate. Glycine equivalents are set at 25 g/kg because inclusions of up to 20 g/kg have been recommended (Siegert and Rodehutscord, 2019). The basic parameters would include growth performance, relative abdominal fat-pad weights, digestive dynamics of starch and protein (N), apparent ileal digestibility coefficients of amino acids, plasma concentrations of NH_3_ and free amino acids. Additionally, parameters for acid-base balance, concentrations of uric acid in excreta, and glutamine concentrations in skeletal muscle tissue should provide useful data. Concentrations of 3-methylhistidine in systemic plasma as a biomarker for protein turnover (Kochlik et al., 2018) could be used to assess the possible anabolic properties of glutamine. This evaluation could identify appropriate inclusion levels of glutamine, glutamate and glycine equivalents on a collective basis in reduced-CP broiler diets. The outcomes of the proposed study should declare if glutamine does, in fact, hold potential in reduced-CP diets for broiler chickens.

## Author contributions

The concept of this perspective was advanced by **Peter H. Selle** and **Shemil P. Macelline**. The first draft was written by **Peter H. Selle** and **Shemil P. Macelline**. And, **Sonia Yun Liu** and **Mehdi Toghyani** were responsible for reviewing and editing the first draft and all co-authors contributed to the final compilation and revisions of this review.

## Declaration of competing interest

We declare that we have no financial and personal relationships with other people or organizations that can inappropriately influence our work, and there is no professional or other personal interest of any nature or kind in any product, service and/or company that could be construed as influencing the content of this paper.
